# Methylation patterns associated with C-reactive protein in racially and ethnically diverse populations

**DOI:** 10.1080/15592294.2024.2333668

**Published:** 2024-04-03

**Authors:** Jessica I. Lundin, Ulrike Peters, Yao Hu, Farah Ammous, Christy L. Avery, Emelia J. Benjamin, Joshua C. Bis, Jennifer A. Brody, Chris Carlson, Mary Cushman, Chris Gignoux, Xiuqing Guo, Jeff Haessler, Chris Haiman, Roby Joehanes, Silva Kasela, Eimear Kenny, Tuuli Lapalainien, Daniel Levy, Chunyu Liu, Yongmei Liu, Ruth J.F. Loos, Ake Lu, Tara Matise, Kari E. North, Sungshim L. Park, Scott M. Ratliff, Alex Reiner, Stephen S. Rich, Jerome I. Rotter, Jennifer A. Smith, Nona Sotoodehnia, Russell Tracy, David Van den Berg, Huichun Xu, Ting Ye, Wei Zhao, Laura M. Raffield, Charles Kooperberg, On Behalf of the PAGE Study

**Affiliations:** a Division of Public Health Sciences, Fred Hutchinson Cancer Center, Seattle, WA, USA; b Department of Epidemiology, School of Public Health, University of Michigan, Ann Arbor, MI, USA; c Department of Genetics, University of North Carolina, Chapel Hill, NC, USA; d Boston Medical Center, Boston University Chobanian and Avedisian School of Medicine, Boston University School of Public Health, Boston, MA, USA; e Cardiovascular Health Research Unit, Department of Medicine, University of Washington, Seattle, WA, USA; f Department of Medicine, Larner College of Medicine at the University of Vermont, Burlington, VT, USA; g Interdisciplinary Quantitative Biology, University of Colorado, Boulder, CO, USA; h The Institute for Translational Genomics and Population Sciences, Department of Pediatrics, The Lundquist Institute for Biomedical Innovation at Harbor-UCLA Medical Center, Torrance, CA, USA; i Department of Environmental Medicine and Public Health, Keck School of Medicine, University of Southern California, Los Angeles, CA, USA; j Population Sciences Branch, National Heart, Lung, and Blood Institute of the National Institutes of Health, Bethesda, MD, USA; k New York Genome Center, New York, NY; l Icahn School of Medicine at Mount Sinai, New York, NY, USA; m Department of Biostatistics, Boston University School of Public Health, Boston, MA, USA; n Duke Molecular Physiology Institute, Duke University, Durham, NC, USA; o Department of Human Genetics, University of California LA, Los Angeles, CA, USA; p Department of Genetics, Rutgers University, New Brunswick, NJ, USA; q Epidemiology Program, University of Hawaii Cancer Center, Honolulu, HI, USA; r Department of Epidemiology, University of Washington, Seattle, WA, USA; s Center for Public Health Genomics, University of Virginia, Charlottesville, VA, USA; t Department of Epidemiology, School of Public Health, and Survey Research Center, Institute for Social Research, University of Michigan, Ann Arbor, MI, USA; u Cardiovascular Health Research Unit, Harborview Medical Center, Seattle, WA, USA; v Department of Biochemistry, University of Vermont, Burlington, VT, USA; w Division of Endocrinology, Diabetes and Nutrition, Department of Medicine, University of Maryland School of Medicine, Baltimore, MD, USA; x Department of Biostatistics, School of Public Health, University of Washington, Seattle, WA, USA; y Survey Research Center, Institute for Social Research, University of Michigan, Ann Arbor, MI, USA

**Keywords:** Inflammation, C-reactive protein, methylation, epigenetics, EWAS, racial and ethnic diversity, Mendelian randomization, causal pathway

## Abstract

Systemic low-grade inflammation is a feature of chronic disease. C-reactive protein (CRP) is a common biomarker of inflammation and used as an indicator of disease risk; however, the role of inflammation in disease is not completely understood. Methylation is an epigenetic modification in the DNA which plays a pivotal role in gene expression. In this study we evaluated differential DNA methylation patterns associated with blood CRP level to elucidate biological pathways and genetic regulatory mechanisms to improve the understanding of chronic inflammation. The racially and ethnically diverse participants in this study were included as 50% White, 41% Black or African American, 7% Hispanic or Latino/a, and 2% Native Hawaiian, Asian American, American Indian, or Alaska Native (total *n* = 13,433) individuals. We replicated 113 CpG sites from 87 unique loci, of which five were novel (*CADM3*, *NALCN, NLRC5, ZNF792*, and cg03282312), across a discovery set of 1,150 CpG sites associated with CRP level (*p* < 1.2E–7). The downstream pathways affected by DNA methylation included the identification of *IFI16* and *IRF7* CpG-gene transcript pairs which contributed to the innate immune response gene enrichment pathway along with *NLRC5*, *NOD2*, and *AIM2*. Gene enrichment analysis also identified the nuclear factor-kappaB transcription pathway. Using two-sample Mendelian randomization (MR) we inferred methylation at three CpG sites as causal for CRP levels using both White and Black or African American MR instrument variables. Overall, we identified novel CpG sites and gene transcripts that could be valuable in understanding the specific cellular processes and pathogenic mechanisms involved in inflammation.

## Introduction

Inflammation is a complex immune response including not only a rapid response to injury and pathogens, but also contributes to the pathophysiology of chronic diseases such as cardiovascular disease, cancer, and diabetes [1–3]. The initial inflammatory response involves the release of interleukin (IL)-6 into circulation. The classic signalling activities include IL-6 signalling the synthesis of C-reactive protein (CRP) in the liver. CRP is readily measured in the blood and is commonly used as a biomarker of inflammation [4]. This biomarker can be extremely high after injury or in response to a pathogen (e.g., >10 mg/L), however moderate-levels (e.g., > 3 mg/L) measured in blood indicate chronic low-level inflammation related to risk of disease [5]. Even with the high sensitivity of CRP measurements, and its use as an indicator of increased chronic disease risk, CRP is not itself the causal factor and the precise roles of inflammation in the development and progression of disease are not known [4]. Two genome-wide association studies (GWAS) in European ancestry populations identified candidate loci of genetic variants associated with CRP enriched for immune pathways, liver metabolic pathways, and chronic obstructive pulmonary disease, with the strongest association for variants at the *CRP* and *IL-6 R* locus [6,7]. However, there was not a single trait associated variant at either of these loci. In all, the lead variants at all distinct loci explained ~7% [6] and ~16% [7] of the CRP variance.

DNA methylation may contribute to the variation in disease phenotype biomarkers and mediate the effects of genetic and environmental factors on markers of chronic inflammation. DNA methylation is an epigenetic modification characterized by the addition of methyl groups predominantly to cytosines at CpG sites (regions in the DNA sequence where cytosine is followed by guanine from the 5’ to 3’ direction) and plays a pivotal role in gene expression through promoter silencing [8]. DNA methylation has been linked to CRP inflammatory marker in previous studies, for example methylation of sites within *AIM2, PHOSPHO1*, and *SOC3* genes [9,10]. Methylation pattern differences and associated disease risk across race and ethnic groups has been reported, including for lupus, an autoimmune disease [11–14]; but, there are major gaps in diversity in epigenome-wide studies of inflammatory biomarkers with a predominance of studies performed in European ancestry populations (White race and ethnicity group designation) [9,10,15–17]. The importance of this gap in diversity is further highlighted by previously established differences in CRP levels between race and ethnic groups; in particular, CRP concentration is nearly two times greater in Black than White individuals, even after accounting for other factors, a difference that remains unexplained [18]. Above this, studies show differences in the epidemiology and features of inflammation-related disease, including cardiovascular disease, in patients of different race and ethnicity [19,20]. As such, broadening the understanding of inflammation-related differences in methylation patterns across race and ethnic groups may improve disease detection and progression across a more diverse population.

In the current study, we sought to improve the understanding of the genetic and epigenetic mechanisms underlying the variation of markers of inflammation by: (1) identifying novel CpG sites associated with inflammation (CRP) in a discovery sample of 10,329 ancestrally diverse participants, with significant CpG sites carried forward to an independent sample of 3,104 participants for replication; (2) assessing the downstream pathways affected by DNA methylation and functional enrichment of these genes; and (3) identifying CpG sites that support inference of a causal effect on CRP levels.

## Materials and methods

### Study populations

A total of nine studies comprising over 13,000 participants were included in the analysis. Five studies were included in the discovery stage for association of methylation (CpG) site and CRP: the Atherosclerosis Risk in Communities (ARIC) study (*n* = 2,311), four Women’s Health Initiative (WHI) ancillary substudies [EMPC (AS315), BA23, AS311, and LLS] (*n* = 3,886), the Jackson Heart Study (JHS) (*n* = 1,323), the Multiethnic Cohort study (MEC) (*n* = 265), and the Framingham Heart Study (FHS) (*n* = 2,544). Four studies were included in the replication stage: the Amish study (AMISH) (*n* = 288), the Cardiovascular Health Study (CHS) (*n* = 734), the Genetic Epidemiology Network of Arteriopathy (GENOA) (*n* = 1,202), and the Multi-Ethnic Study of Atherosclerosis (MESA) (*n* = 880). Self-reported Black or African American, Hispanic or Latino/a, and White participants formed the three largest self-identified race and ethnicity groups. Smaller numbers of Japanese American, Chinese American, Native Hawaiian, American Indian, and Alaska Native participants were also represented. All studies obtained written informed consent from participants and were approved by local institutional review boards and ethics committees. Additional information on participating studies is detailed in the supplemental material.

### Inflammation marker measurements

CRP (mg/L) was measured using high-sensitivity assays in all studies. Study specific methods on quantification and details of specific assays are listed in the supplemental material (Table S1). CRP and DNA methylation quantification were measured in blood samples from the same examination (ARIC, JHS, MEC, FHS, MESA) or samples collected within one year (WHI), except for the GENOA and AMISH studies which had up to a 7-year time lapse between the two measurements and were both in the replication stage (Table S1). CRP values were natural log transformed. Participants with log blood levels outside of four standard deviations from the median were excluded from the analysis.

### DNA methylation measurement, quality control, and normalization

DNA methylation was quantified in each participating study independently. Levels were measured from peripheral blood leukocytes (‘buffy coat’) isolated from whole blood in all studies. DNA methylation was measured using the Illumina Infinium HumanMethylation450 BeadChip (HM450) in ARIC, FHS, AMISH, GENOA, and CHS studies, and three of the WHI ancillary studies [EMPC(AS315), BAA23, AS311]. The Illumina Infinium MethylationEPIC BeadChip (EPIC) was used in WHI (LLS substudy), JHS, GENOA, MEC, and MESA studies; only CpG sites also reported for the HM450 chip were retained. Each cohort conducted their own quality control to filter the DNA methylation data on marker (probe) and sample call rate thresholds, and normalization to reduce unwanted technical variation (Table S1). A M-value was calculated for each CpG site representing the log2 ratio of the intensities of methylated versus unmethylated probes at that site. Houseman and other methods were implemented to estimate white blood cell proportions (CD8T, CD4T, natural killer, B-cell, monocyte, and granulocyte) (Table S1) [21]. To avoid spurious signals in DNA methylation data, we excluded probes that co-hybridize to alternate genomic sequences or overlapped with genomic variations (i.e., SNP-introduced artefact) [22]. Additional CpG site exclusions were applied for probes shown to have SNP-introduced artefact or MAF < 1% in race and ethnic population groups (population-specific probe information available for White and Black or African American only) [22]. After quality control, and restriction to autosomal chromosomes, 394,880 probes remained and were examined.

### EWAS, genomic control, and meta-analysis

**Figure 1.**
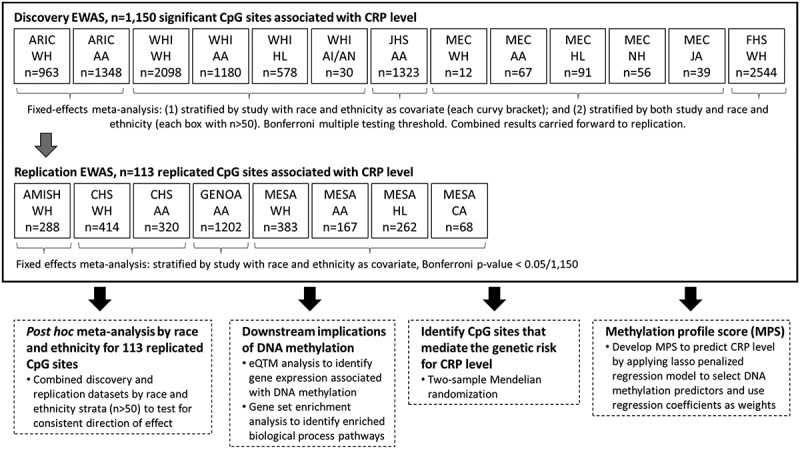
Summary of the overall study design. Main analysis (EWAS) in solid-line box, showing discovery and replication studies. Additional analyses performed on the replicated CpG sites described in dashed-line boxes.

We performed an epigenome-wide association analysis (EWAS) of CRP using linear mixed effects models (‘nlme’ package in R) separately in each cohort or ancillary study. To evaluate significant CRP-associated methylation markers across racially and ethnically diverse participants, discovery models were analysed in two ways: (1) stratified by study with self-identified race and ethnicity included as a covariate and (2) stratified by both study and race and ethnic group (Figure 1). Both the combined and race stratified models were evaluated to ensure any race specific CpG sites would be carried through to the replication stage. Participants were excluded in the race and ethnicity adjusted models when <10 were available in a race and ethnic group, and excluded from the race and ethnicity stratified model when <50 were available in a race and ethnic group. The CRP inflammation trait was tested for association with each CpG site M-value adjusted for age, sex, body mass index (BMI), smoking (current, former, never), race and ethnic group (in the adjusted models), and study-specific covariates (if applicable). White blood cell proportions, the first 10 genetic principal components (PCs) (generated by study), and chip row and column (technical covariates) were also included in the models as fixed-effects, and chip number was adjusted as a random-effect covariate. All summary statistics were adjusted for bias and inflation to prevent reporting false-positive signals due to bias and unwanted variation such as population stratification; we applied this genomic control using the ‘BACON’ package in R [23] (Table S2). Results were combined through fixed-effect inverse-variance weighted meta-analysis using METAL [24]. Meta-analyses were run separately by race and ethnicity adjusted models, and the stratified models. Bonferroni significance thresholds were used for these discovery datasets (*p* < 0.05/394,880), with an additional test of heterogeneity applied to the race and ethnicity stratified models’ meta-analysis (p-value of heterogeneity based on Cochran Q test, *p* > 0.05/number of significant CpG sites). The CpG sites identified in the adjusted and stratified discovery sets were combined and carried forward for replication. Replication models were analysed as race and ethnicity adjusted linear mixed effects models, following all processing steps described above for the discovery data sets. The race and ethnicity adjusted replication datasets were tested with the Bonferroni significance threshold based on the number of CpG sites identified in the discovery datasets. We applied the ‘gaphunter’ R function in the minfi package to the beta values for two of the replication datasets, MESA and CHS, to rule of any residual influence from genetic variation among the 1,150 discovery set CpG sites in these racially and ethnically diverse cohorts [25]. We used a 0.05 threshold and cut-off value of 0.01.

We tested for consistent direction of effect across race and ethnic strata for the replicated CpG sites to demonstrate the robustness of findings across populations. *Post hoc* meta-analyses were performed separately by race and ethnicity (combining discovery and replication datasets, when >50 participants were available in a race and ethnic group) (Figure 1). Race and ethnicity-specific meta-analyses were performed in Black or African American, Hispanic or Latino/a, and White participants, the three largest race and ethnic groups, following the same analysis plan as above (without the adjustment for race and ethnicity).

### Independent loci and annotation of CpG sites

We used the genome coordinates provided by Illumina (GRCh37/hg19) to identify independent loci. A distance criterion of 1 Mb on either side of each epigenome-wide significant signal was used to define independent loci followed by selection of the CpG site with the lowest p-value. In addition to the annotation of proximal gene to the CpG site provided by Illumina based on RefSeq database, a literature search and cross reference with the EWAS Catalog (described below) [26] was conducted to further annotate the CpG sites to proximal genes.

### Downstream pathways affected by DNA methylation: expression quantitative trait methylation (eQTM)

The replicated CpG sites associated with CRP were further evaluated for expression quantitative trait methylation (eQTM) analysis to identify downstream effects of DNA methylation on gene expression. eQTM lookups were based on previous analysis of the FHS participants using DNA methylation data and RNA sequencing data to quantify the association between DNA methylation and gene expression adjusted for age, sex, white blood cell count, blood cell fraction, platelet count, genetic PCs, and DNA methylation PCs [27]. We identified significant *cis* associations between CpG sites and gene transcripts where the CpG site was within 1Mb of the transcription start site (p-value of 1.0 × 10^−7^) and *trans* associations where the CpG site was greater than 1Mb of the transcription start site (stricter p-value of 1.0 × 10^−14^ to reduce noise and false positives).

### Identify biological process pathways affected by DNA methylation: Gene Ontology (GO) pathway enrichment analysis

To analyse the differentially methylated genes for functional enrichment, Gene Ontology (GO) for Biological Process was performed using the R package ‘clusterProfiler’ [28,29]. All replicated CpG sites for association with CRP were first evaluated using the proximal genes to identify enriched GO biological pathways. For this analysis, the annotations of CpG sites to the corresponding proximal genes was performed using the ‘missMethyl’ R package [30]. For all significant *cis*-eQTM CpG-transcript pairs, we conducted a second GO pathways analysis of the gene transcripts to identify biological pathways that may be affected by DNA methylation patterns associated with serum CRP level. For both analyses, the enrichment result differences with a false discovery rate (FDR) p-value of *p* < 0.05 were retained. These results were further reduced using the treeplot function within the enrichplot R package which performs functional groupings for enrichment results of gene set enrichment analyses [31]. The GO pathway with the lowest p-value in each grouping was selected. Kyoto Encyclopedia of Genes and Genomes (KEGG) pathways analyses were also performed to better understand the functional pathways of genes proximal to the identified CpG sites and for *cis-*eQTM gene transcript pairs. KEGG pathway analysis was performed using the DAVID database (https://david.ncifcrf.gov/) [32].

### Mendelian randomization to evaluate causal pathway

A two-sample Mendelian randomization (MR) test of causation was performed to infer whether DNA methylation was causal or consequential to CRP inflammation biomarker level [33]. The instrument variables (IV) were: (1) single nucleotide polymorphisms (SNPs) associated with methylation values from methylation quantitative trait loci (mQTLs) analyses (exposure IV) and (2) SNPs associated with CRP from genome-wide association studies (GWAS) (outcome IV); each calculated from separate studies and described below. The test of causation was performed to evaluate DNA methylation as causal for a change in CRP level with individual models for each CpG site.

We performed mQTL analysis to test the association of SNPs within 1 Mb of each of the replicated CpG sites (known as *cis*-mQTLs) in WHI and ARIC participants with both genetic and DNA methylation data (74% overlap with participants included in the EWAS). The mQTLs were calculated separately for White and Black or African American race and ethnic groups; other race groups were not included due to smaller sample size. Matrix eQTL software [34] was used for preliminary association analysis of SNPs with CpG sites (M-value) in the HM450 array adjusted for the first five genetic ancestry principal components, age, bead, chip, row, column, smoking, BMI, sex (ARIC only), white blood cell type proportions, and study centre (ARIC only). Only mQTLs calculated from SNPs with a minor allele frequency greater than 1% and with significant SNP-CpG site associations (*p* < 0.001) were retained. The mQTLs were further pruned by linkage disequilibrium (LD) correlation using 1000 Genomes Project populations as the reference (EUR for White and AFR for Black or African American). To select the independent IVs for each CpG site, the SNPs were grouped by LD (r2 < 0.1 for SNPs within 1 Mb genomic region) and the SNP with the lowest P-value per group was retained. An F-statistic was calculated each for Exposure IV (beta^2^/standard error^2^) to test instrument strength [35]. We used genetic association data for the SNP-inflammation biomarker association from recent GWAS data evaluating SNP associations with CRP level (ukb-d-30710_raw; UK Biobank, Neale Lab) [6] available through MR-Base, a database of published GWAS available for MR (www.mrbase.org) [33]. Harmonization of the exposure IV and outcome IV datasets retained SNPs that were available from both. The number of exposure and outcome instrument variables before and after LD clumping and available for harmonization of final data for each MR, along with number of represented CpG sites, is available in supplemental material (Table S3).

The analysis was performed using various MR methods available in the ‘TwoSampleMR’ package in R [33] where each method provided an estimate of the causal effect of exposure on the outcome. If there was only one remaining SNP, we used Wald ratio test to assess the causal effect of SNP methylation. If there were two or more remaining SNPs, the analysis was performed using the inverse variance-weighted (IVW) method which utilized the combined ratio estimates from each SNP into an overall estimate [36]. A Weighted Median (WM) Estimator was also used, which provides a consistent estimate even when up to 50% of the IVs are invalid [37]. We performed sensitivity analysis for horizontal pleiotropy by MR Egger’s regression [38], and applied a Cochran’s Q test for heterogeneity. We also applied the Robust Adjusted Profile Score (MR.RAPS) method available in the ‘MR.RAPS’ package in R [35], which is robust to systematic and idiosyncratic pleiotropy, and can give robust inference for MR analysis in the presence of many weak instruments. The MR.RAPS corrects for pleiotropy using robust adjusted profile scores and is particularly recommended when the exposure and outcome are complex traits [39]. MR.PRESSO method was applied to evaluate horizontal pleiotropy and test for outliers [40], and the leave-one-out analysis was performed to identify if a single SNP was driving the association. MR analysis was run twice, each using the White and Black or African American mQTL data harmonized with the UK Biobank GWAS data, and performed iteratively through all selected CpG sites. The CpG sites that were significant for Wald ratio, IVW, or WM test (*p* < 0.1) in either the White or the Black or African American MR were retained and further evaluated for weak IVs (F-statistic <10) or outlier variants identified by MR-PRESSO outlier test. Last, MR Steiger was performed on the final list of CpG mediators to infer the direction of the causal effect by testing if the genetic instruments predicted the outcomes more than the exposures by comparing the variance in exposures and outcomes explained by each genetic variant (r2) [41]. Final retained CpG sites had a FDR < 0.05 for the IVW analysis and maintained a consistent direction with the EWAS results.

### Methylation profile score (MPS) to predict CRP level

A methylation profile score (MPS) for CRP level prediction was developed using the significant CpG sites identified in the EWAS discovery set (*n* = 1,150 CpG sites) applied first to the MESA replication cohort (training) and then tested on the CHS replication cohort. A lasso penalized regression model was run using the ‘glmnet’ function in R to train DNA methylation predictors with CRP level as the outcome. We applied tenfold cross-validation, and the mixing parameter was set to 1 for our lasso penalty. The training set was the DNA methylation beta values from the MESA dataset with age, sex, race, BMI, smoking, WBCs, genetic PCs, and technical covariates included without penalty. The lasso regression coefficients from the resulting 38 selected CpG sites were applied as weights to residuals of the DNA methylation values in CHS, the test dataset, after regressing out age, sex, race, BMI, smoking, WBCs, genetic PCs, and technical covariates (random effects). The sum of these 38 values was considered the MPS. Using a mixed effect linear regression model, we calculated the CRP variance explained by the methylation score (‘rsq’ package in R) in a univariate analysis, and adjusted for age, sex, race, BMI, smoking, WBCs, and genetic PCs with technical covariates included as a random effect.

### Cross-reference with the EWAS Catalog to evaluate biologic plausibility

For CpG sites identified as significantly associated with CRP, and for sites identified in the MR analyses, we looked for previously known trait associations in the EWAS Catalog [26]. This catalogue contains CpG-trait associations from published EWAS studies to query EWAS associations quickly and easily. This provides the opportunity to gain insight into the molecular underpinnings of disease as well as the impact of traits and exposures on the DNA methylome.

## Results

### Discovery and replication study populations

This study included 13,433 participants from nine independent studies. The characteristics of the five discovery cohorts (*n* = 10,329) and four replication cohorts (*n* = 3,104) are presented in Table 1. The race and ethnic groups included White participants from WHI, ARIC, MEC, and FHS in the discovery set, and AMISH, CHS, and MESA in the replication set; Black or African American participants in the ARIC, WHI, JHS, and MEC discovery sets, and CHS, GENOA, and MESA in the replication set; and Hispanic or Latino/a from WHI and MEC in the discovery set, and MESA in the replication set. In addition, the discovery set included American Indian or Alaska Native (WHI), Native Hawaiian (MEC), and Japanese American (MEC) participants, and the replication set included Chinese American (MESA) participants. The mean age in the participating studies ranged from 57 y in the JHS, ARIC, and AMISH cohorts to 70 y in the WHI cohort (LLS substudy). The mean BMI ranged from 24 to 32 kg/m^2^. The majority (~72%) of the samples were from women due to the sizable contribution of participants from WHI, an all women study. Mean serum CRP levels ranged from 0.8 mg/L in the MEC Japanese American participants to 3.7 mg/L in the WHI American Indian or Alaska Native participants (Table 1).

**Table 1.** Characteristics of the discovery (*n* = 10,329) and replication (*n* = 3,104) study populations.

Study	Chip	Race and ethnicity	Age, yrs; mean (sd)	BMI, kg/m^2^; mean (sd)	Smoking; % c/f/n or % cy/cn^a^	n (% F)^b^	ln CRP, mg/L; mean (sd)
*Discovery*							
ARIC	HM450	WH	60 (5)	26 (4)	18/39/42	963 (59)	0.8 (1.1)
		AA	57 (6)	30 (6)	22/31/46	1,348 (65)	1.2 (1.2)
WHI	HM450/	WH	70 (9)	28 (6)	8/38/54	2,098 (100)	0.8 (1.1)
	EPIC	AA	65 (9)	31 (6)	12/39/48	1,180 (100)	1.2 (1.2)
		HL	63 (8)	30 (6)	7/30/62	578 (100)	1.1 (1.0)
		AI/AN	64 (8)	31 (6)	9/34/58	30 (100)	1.3 (1.1)
JHS	EPIC	AA	57 (12)	32 (7)	14/22/64	1,323 (62)	0.9 (1.2)
MEC	EPIC	WH	72 (8)	24 (4)	100/0/0	12 (73)	0.9 (1.3)
		AA	66 (7)	27 (6)	100/0/0	67 (67)	0.8 (1.1)
		HL	68 (7)	26 (4)	100/0/0	91 (42)	1.0 (1.1)
		NH	66 (6)	26 (4)	100/0/0	56 (55)	0.4 (1.4)
		JA	63 (7)	25 (4)	100/0/0	39 (29)	−0.2 (1.5)
FHS	HM450	WH	66 (9)	28 (5)	9/91	2,544 (54)	0.5 (1.1)
*Replication*							
AMISH	HM450	WH	57 (14)	28 (5)	9/16/75	288 (54)	0.8 (1.3)
CHS	HM450	WH	75 (5)	27 (5)	9/44/46	414 (61)	1.0 (1.2)
		AA	73 (5)	29 (5)	16/38/46	320 (63)	1.2 (1.1)
GENOA	HM450	AA	67 (7)	31 (7)	11/32/58	271 (82)	1.3 (1.1)
	EPIC	AA	63 (10)	32 (7)	13/27/60	931 (72)	1.2 (1.1)
MESA	EPIC	WH	61 (10)	28 (5)	13/45/42	383 (51)	0.5 (1.1)
		AA	61 (10)	31 (6)	20/33/47	167 (57)	0.8 (1.1)
		HL	59 (9)	30 (5)	11/34/54	262 (55)	0.8 (1.1)
		CA	61 (10)	25 (3)	7/22/71	68 (46)	0.2 (1.1)

Abbreviations: AA, Black or African American; AI/AN, American Indian or Alaska Native; AMISH, Amish study; ARIC, Atherosclerosis Risk in Communities; CHS, Cardiovascular Health Study; CRP, C-reactive protein; CA, Chinese American; FHS, Framingham Heart Study; GENOA, Genetic Epidemiology Network of Arteriopathy; HL, Hispanic or Latino/a; JA, Japanese American; JHS, Jackson Heart Study; MEC, Multiethnic Cohort study; MESA, Multi-Ethnic Study of Atherosclerosis; NH, Native Hawaiian; WH, White; WHI, Women’s Health Initiative.

^a^c/f/n = current/former/never; cy/cn = current, yes/current, no.

^b^% F (female).

### Epigenome-wide association study (EWAS) of CRP

We performed an EWAS of CRP using linear mixed effects models separately in each cohort. To evaluate significant CRP-associated methylation markers across racially and ethnically diverse participants, discovery models were meta-analysed separately by: (1) race and ethnicity adjusted (*n* = 1060) and (2) stratified (re-combining all race and ethnic groups and testing for heterogeneity) models (*n* = 1046, including 90 CpGs not identified in the race adjusted analysis) (Figure 1). The results of each analysis were combined and carried forward for replication. In total, we identified 1,150 CpG sites significantly associated with CRP in the discovery meta-analyses (Figure 1; Table S4). The beta coefficients from the mixed effects models were highly correlated (Pearson correlation coefficient, 0.98) when restricting the discovery sets to individuals with CRP level less than 10 mg/dL. This supports that these sites represent chronic low-grade inflammation.

**Figure 2.**
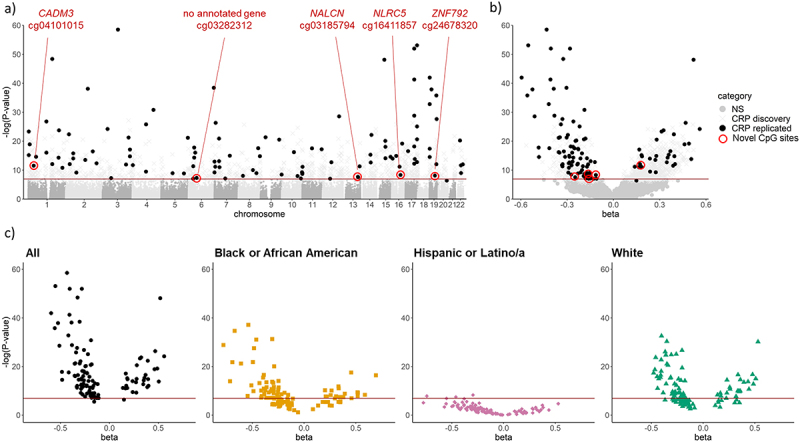
CpG sites significantly associated with CRP; (a) Manhattan and (b) volcano plots of 1,150 significant CpG sites in the discovery set (CRP discovery), and 113 replicated CpG sites (CRP replicated) with a red circle around the five novel CpG sites; (c) volcano plots of replicated CpG sites with *post hoc* meta-analysis performed separately by race and ethnic groups to demonstrate the consistent direction of effect [NS = not significant].

The CRP replication meta-analysis of 3,104 individuals replicated 113 CpG sites (*p* < 0.05/1,150); 107 of these were maintained (*p* < 0.05) when restricting the replication to cohorts (MESA, CHS) with paired methylation and CRP measurements (Table S4). CpG sites associated with CRP were distributed across the genome (Figure 2a). Increased CRP level was associated with hyper-methylation at 30 CpG sites and hypo-methylation at 83 CpG sites (Figure 2b). The 113 replicated CpG sites identified in the main analysis were robust across populations, as demonstrated by a consistent direction of association in the *post hoc* race and ethnicity stratified analysis (Figure 2c; Table S4). Only one CpG site varied in direction of association (cg02417857, *SOX2OT*), showing a negative direction of effect in the Hispanic or Latino/a race and ethnic strata and a positive direction in the White and Black or African American strata and race adjusted analyses. Of the 1,037 CpG sites that were significant in the discovery meta-analysis only, 94% (*n* = 979) reported consistent direction of association across the *post hoc* race and ethnicity stratified analysis suggesting that many of the non-replicated results may be true associations. We applied the Gaphunter function to all 1,150 discovery CpG sites to identify CpG probes confounded by adjacent SNPs and found one replicated CpG site (cg27307975 near *LIPC*) exhibited weak evidence of confounding. The 113 replicated CpG sites included five novel, independent sites across five chromosomes: cg04101015, *CADM3*; cg03185794, *NALCN*; cg16411857, *NLRC5*; cg03282312, not proximal to any known coding sequence; and cg24678320, *ZNF792* (Figure 2a,b). The remaining 108 sites were previously reported in the literature (Table S4).

### Downstream pathways affected by DNA methylation: eQTM and pathway enrichment analysis

CpG-gene transcript pairs (*cis-*eQTM analysis) were analysed to characterize the association of DNA methylation and gene expression. The 113 CpG sites evaluated for eQTM analysis to identify downstream effects of DNA methylation on gene expression had 51 *cis*-eQTM transcript matches (i.e., associations where the CpG site was within 1Mb of the transcription start site, p-value <1.0 × 10^−7^) across 32 of the 113 CpG sites (1–6 gene transcripts for each CpG site) (Table 2; Table S5). The remaining CpG sites did not have significant *cis*-eQTM transcript-pairs. There were 645 *trans-*QTM transcript matches (i.e., associations where the CpG site was >1Mb of the transcription start site, p-value <1.0 × 10^−14^) across 52 of the 113 CpG sites (1–304 gene transcripts for each CpG sites). The remaining CpG sites did not have significant *trans*-eQTM transcript-pairs.

**Table 2.** CpG-gene transcript pairs; *cis-*eQTM analysis results (1 × 10^−7^) for CpG sites significantly associated with CRP; see table S5 for full list of eQTM results.

CpG site	proximal gene	beta	se	log10 pval	*cis-*eQTM associated gene transcript(s)^b^	beta	se	log10 pval
cg16411857	*NLRC5*	−0.11	0.02	−4.80	NLRC5*, MT2A, NUP93	−1.67	−12.82	−35.54
cg10636246	*AIM2*	−0.33	0.02	−10.38	AIM2*, IFI16	−2.48	−12.53	−34.04
cg23570810	*IFITM1*	−0.15	0.03	−6.04	IFITM1*, IRF7, AC136475.2, AC136475.7	−1.80	−11.48	−28.88
cg14965639	*STON1-GTF2A1L*	−0.21	0.03	−5.32	*STON1*	1.48	11.18	−27.49
cg10552523	*IFITM1*	−0.19	0.03	−8.35	IFITM1*, IRF7, AC136475.2, AC136475.7	−2.48	−10.71	−25.37
cg06051311	*TRIM15*	−0.11	0.02	−4.85	AL645929.3*, HLA-K, HCG4B, AL645929.1	2.45	9.47	−20.14
cg23570810	*IFITM1*	−0.15	0.03	−6.04	TALDO1*, RPLP2	0.60	9.27	−19.35
cg03998636	*RAB20*	−0.33	0.05	−5.97	*RAB20*	−3.31	−9.16	−18.93
cg01243823	*NOD2*	−0.17	0.03	−5.61	SNX20*, NOD2, AC007728.2	−0.83	−9.03	−18.40
cg08548559	*PIK3IP1*	−0.24	0.03	−4.80	*RNF185*	0.80	8.57	−16.69
cg19821297	no gene^a^	−0.52	0.04	−8.26	DNASE2*, CALR	−1.74	−8.41	−16.11
cg06092244	*STON1-GTF2A1L*	−0.20	0.02	−5.43	*STON1*	1.80	8.30	−15.73
cg08548559	*PIK3IP1*	−0.24	0.03	−4.80	PISD*, PIK3IP1, SFI1	−1.05	−7.55	−13.19
cg24678320	*ZNF792*	−0.16	0.03	−5.74	ZNF30*, AC008555.7	−1.17	−7.50	−13.02
cg25392060	no gene	0.39	0.05	−4.53	*LINC01300*	0.55	7.34	−12.51
cg08423142	*MYO1E*	−0.26	0.03	−4.48	*MYO1E*	−2.73	−7.16	−11.95
cg22304262	*SLC1A5*	−0.33	0.03	−5.58	*SLC1A5*	−1.27	−6.76	−10.74
cg18181703	*SOCS3*	−0.55	0.04	−18.15	*CYTH1*	0.49	6.65	−10.42
cg16936953	*TMEM49*	−0.41	0.03	−21.29	TMEM49*, DHX40	0.43	6.52	−10.05
cg09182678	no gene	−0.16	0.02	−4.88	*BX539320.1*	−2.23	−6.29	−9.42
cg18942579	*TMEM49*	−0.41	0.03	−13.97	*TMEM49*	0.47	6.28	−9.40
cg18181703	*SOCS3*	−0.55	0.04	−18.15	*SOCS3*	−1.89	−6.25	−9.31
cg10922280	*DPEP2*	0.49	0.05	−6.16	*DPEP2*	−1.38	−6.20	−9.15
cg26470501	*BCL3*	−0.56	0.04	−9.82	*IGSF23*	1.06	6.16	−9.06
cg12054453	*TMEM49*	−0.28	0.02	−22.49	*DHX40P1*	−0.27	−6.11	−8.93
cg10552523	*IFITM1*	−0.19	0.03	−8.35	*TALDO1*	0.59	6.11	−8.91
cg17501210	*RPS6KA2*	−0.30	0.02	−6.37	*RNASET2*	−1.34	−6.01	−8.67
cg01409343	*TMEM49*	−0.38	0.03	−13.15	*TMEM49*	0.57	5.98	−8.59
cg25392060	no gene	0.39	0.05	−4.53	*PTP4A3*	−1.97	−5.91	−8.40
cg15551881	*TRAF1*	0.28	0.03	−4.41	*CUTALP*	3.27	5.85	−8.24
cg18942579	*TMEM49*	−0.41	0.03	−13.97	*DHX40P1*	−0.30	−5.83	−8.19
cg06051311	*TRIM15*	−0.11	0.02	−4.85	*HLA-U*	−1.03	−5.83	−8.18
cg13823169	no gene	−0.27	0.05	−4.40	*TRAF2*	−0.85	−5.82	−8.17
cg02734358	*GPRIN3*	−0.34	0.03	−9.30	*GPRIN3*	−0.68	−5.78	−8.06
cg18608055	*SBNO2*	−0.60	0.04	−15.52	*POLR2E*	0.77	5.75	−7.98
cg12054453	*TMEM49*	−0.28	0.02	−22.49	*TMEM49*	0.37	5.74	−7.97
cg27023597	*MIR21*	−0.28	0.03	−5.63	*HEATR6*	−0.73	−5.58	−7.57
cg16411857	*NLRC5*	−0.11	0.02	−4.80	*CPNE2*	0.70	5.54	−7.47
cg01409343	*TMEM49*	−0.38	0.03	−13.15	*DHX40P1*	−0.37	−5.53	−7.45
cg24678320	*ZNF792*	−0.16	0.03	−5.74	*ZNF792*	0.71	5.50	−7.38
cg06192883	*MYO5C*	0.52	0.04	−6.28	*FAM214A*	−0.82	−5.47	−7.30
cg02782634	*TMEM49*	−0.23	0.03	−8.92	*TMEM49*	0.62	5.42	−7.18
cg00159243	*SELPLG*	−0.51	0.05	−9.87	*AC007569.1*	1.19	5.35	−7.01

Abbreviations: beta, coefficient for CpG site methylation and gene expression association; se, standard error; log10 pval, log10 p-value for CpG site methylation and gene expression association.

^a^not proximal to any known coding sequence

^b^when number of CpG-gene transcript pairs was greater than one and the eQTM direction of effect was consistent, the beta, se, and -log10pval listed are from the most significant eQTM gene transcript pair (* indicates most significant gene transcript)

To gain mechanistic insight into genes affected by altered DNA methylation status, the differentially methylation genes were evaluated to identify overrepresented GO Biological Process categories and KEGG pathways. The 113 differentially methylated CpG sites associated with CRP were evaluated using the gene proximal to the CpG site. The significant GO enrichment pathways of biological process included: regulation of sulphur metabolic process (GO:0042762; p-value = 9.3 × 10^−6^; geneIDs = *SLC7A11/MIR21/NFE2L2);* and negative regulation of nuclear factor-kappaB (NF-kB) transcription factor activity (GO:0032088; p-value = 3.0 × 10^−5^; geneIDs =* MIR21/BCL3/NOD2/NLRC5/AIM2)* (all FDR <0.05; Table 3; Table S6). The top GO gene enrichment pathways for the *cis-*eQTM transcript pairs for the 113 replicated CpG sites included: positive regulation of innate immune response (GO:0045089; p-value = 1.4 × 10^−7^; geneIDs = *IFI16/IRF7/NOD2/NLRC5/RNF185/AIM2*) and response to interferon-beta (GO:0035256; p-value = 1.9 × 10^−5^; geneIDs *= IFI16/IFITM1/AIM2*) which included negative regulation of NF-kB transcription factor activity (GO:0032088) similar to the proximal gene enrichment pathway analysis (all FDR <0.05; Table 3; Table S6). The 113 replicated CpG sites identified for CRP were analysed in the KEGG pathway which was enriched in proximal genes associated with the tumour necrosis factor (TNF) signalling pathway (*BCL3/NOD2/TRAF1/SOC3*) known to trigger NF-kB, among other integral proteins, that orchestrate the response to inflammation [42] (unadjusted p-value <0.001, FDR > 0.05; Fig. S1). Two KEGG pathways for the *cis-*eQTM gene transcripts were significant with an FDR < 0.05, the cytosolic DNA-sensing pathway (*POLR2E/AIM2/DNASE2/IRF7*) and NOD-like receptor signalling pathway (*TRAF2/AIM2/IFI16/IRF7/NOD2*) (Figs. S2a,b).

**Table 3.** GO biological pathways enriched in genes corresponding to the 113 replicated CpG sites associated with CRP levels, all FDR < 0.05 (see table S6 for full list).

category	GO ID	description	p-value (unadj)	p-value (FDR)	gene
proximal genes	GO:0042762	regulation of sulphur metabolic process	9.3 × 10^−6^	0.015	*SLC7A11/MIR21/NFE2L2*
	GO:0032088	negative regulation of NF-kB transcription factor activity^a^	3.0 × 10^−5^	0.025	*MIR21/BCL3/NOD2/* *NLRC5/AIM2*
*cis-*eQTM transcript pairs	GO:0045089	positive regulation of innate immune response	1.4 × 10^−7^	1.2 × 10^−4^	*IFI16/IRF7/NOD2/NLRC5/RNF185/AIM2*
	GO:0001961	positive regulation of cytokine-mediated signalling pathway	3.1 × 10^−6^	4.8 × 10^−4^	*IRF7/TRAF2/NLRC5/* *RNF185*
	GO:0035456	response to interferon-beta^a^	1.9 × 10^−5^	1.1 × 10^−3^	*IFI16/IFITM1/AIM2*
	GO:0038061	NIK/NF-kappaB signalling	1.1 × 10^−4^	0.006	*PTP4A3/NOD2/TRAF2/* *CALR*
	GO:2000109	regulation of macrophage apoptotic process	1.4 × 10^−4^	0.006	*IRF7/NOD2*

^a^Annotation group includes GO:0032088 Negative regulation of NF-kB transcription factor activity

### Mendelian randomization to evaluate causal pathway

**Figure 3.**
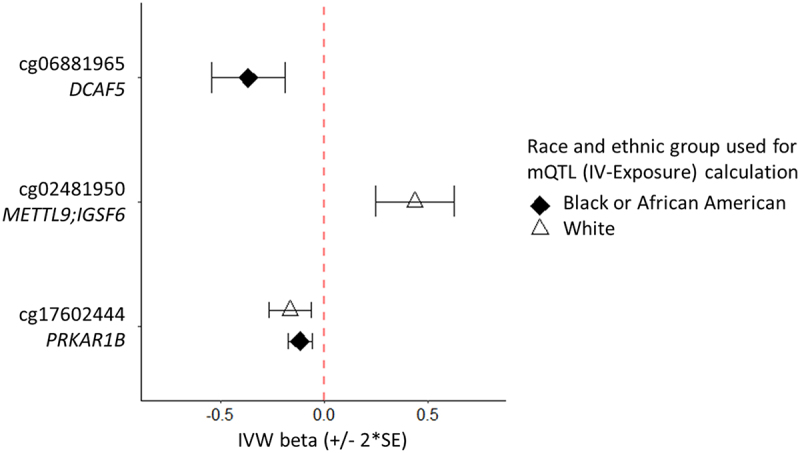
CpG sites inferring DNA methylation as causal to a change in CRP level.

The resulting analyses highlighted methylation as causal for inflammation biomarker level, and in the same direction of effect as the EWAS, associated with three unique CpG sites (*DCAF5*, *METTL9/IGSF6*, *PRKAS1B*; Figure 3; Table S7). These CpG sites met significance using a Bonferroni threshold of mulitple comparisons. One site was significant for both the White (FDR<0.05) and Black or African American (Bonferroni) MR analyses (cg17602444, *PRKAR1B*) . Concerns of weak instrument bias are addressed through testing by various MR method (see methods). All reported associations passed tests of heterogeneity and pleiotropy and were tested for outliers; the F-statistic measure of instrument strength was greater than 10 for all SNPs (Table S3). Further evaluation used MR Steiger, a statistical test to infer the direction of causality between the exposure and outcome under consideration. The Steiger test showed the direction of all causal associations reported as true (Steiger p-value <0.1 × 10^−5^), meaning the genetic instruments explained more variance in the exposure than the outcome and the causal effects reported herewithin are in the correct direction.

### Methylation profile score (MPS) to predict CRP level

The weighted MPS across the 38 CpGs (Table S4) had a mean of 0.0005 and range of −0.73 to 1.18. The score was applied to the CHS test set, explaining 5.1% of the variance in the MPS only model and 22.4% in the fully adjusted model. When removing smoking and BMI the percent variance explained dropped to 10.7%, and the additional removal of age and gender resulted in 10.0% percent variance explained. The MPS in each model above was significantly associated with CRP (all models, *p* < 6 × 10^−9^). The percent variance explained was similar when stratifying the model; the MPS univariate model showed 5.1% variance explained in the Black or African American strata and 5.3% variance explained in the White strata.

## Discussion

We report a large-scale (~13,400 participants) EWAS of CRP including racially and ethnically diverse participants. Meta-analyses revealed 113 CpG-CRP trait associations at 87 independent loci, including five novel CpG sites (proximal to *CADM3*, *NALCN, NLRC5*, and *ZNF792*, and cg03282312). Findings across the 113 CpG sites associated with CRP level support consistent epigenetic associations for inflammation across White, Black or African American, and Hispanic or Latino/a race and ethnic groups. Our methylation profile score, generated using a racially and ethnically diverse population, performed equally across race strata explaining 5% of the CRP variance in both Black or African American and White participants, and over 22% of the CRP variance when adjusting for age, BMI, smoking, and other covariates. We show proximal genes and genesidentified by *cis*-eQTM analysis that are associated with an immune response consistent with inflammation, including the identification of three genes causally associated with CRP levels using both Black or African American and White derived instrumental variables in MR analyses.

A review of the most significant CpG sites highlighted findings consistent with the current literature. The *AIM2, BCL3*, *MYO5C, PHOSPHO1, RPS6KA2, SBNO2, SLC7A11, SOCS3, TMEM49*, and *ZEB2* genes, among others, replicated previously reported CRP-associated CpG sites (Table S4) [9,10]. For the most significant CRP-CpG sites, *SOCS3*, *TMEM49*, and *AIM2*, the EWAS findings of hypomethylation in the Suppressor of Cytokine Signaling (*SOCS*) 3 gene is consistent with previous findings of *SOCS3* as a negative regulator of IL-6 [43], including studies indicating hypomethylation associated with type-2 diabetes [44] and hypertension (specifically, right atrial pressure) [45]. *TMEM49* (transmembrane protein 49), which plays a role in the regulatory process of autophagy, has been associated with inflammation related to Crohn’s disease [46], inflammatory bowel disease [47], and pancreatic cancer [48], among others [9,49]. Lastly, the *AIM2* inflammasome is a multiprotein complex that initiates the innate immune response and contributes to chronic inflammation [50]. There are circumstances where the CpG site proximal to the previously reported gene was associated with a different *cis-*gene. For example, the CpG site cg18608055 (proximal to *SBNO2*) was associated with *POLR2E*, an RNA polymerase associated with cancer (Table 2) which also contributed to the significant KEGG cytosolic DNA-sensing pathway (Fig. S2a) [51,52]. Additionally, cg06192883 (proximal to *MYO5C*) was significantly associated with transcription of *FAM214A*, a gene shown to modify the inflammatory response [53]. These CpG-gene transcripts show biological pathways beyond the most proximal gene.

This study identified five novel CpG sites associated with CRP concentrations. A review of the novel sites using the EWAS Catalog [26] (Table S8) showed previous associations with Crohn’s disease, rheumatoid arthritis, systemic lupus erythematosus, and TNF-α for *NLRC5* (Nod-like receptor family CARD domain containing 5; cg16411857). *NLRC5* modulates inflammatory responses and plays a role in immune diseases including rheumatoid arthritis, as well as liver, renal, heart, lung, and spleen diseases by regulating the NF-kB, type I interferon, and inflammasome signalling pathways [54,55]. Consistent with this, *NLRC5* was associated with the negative regulation of NF-kB transcription factor activity in the GO biological pathway enrichment analysis using both proximal genes and *cis-*eQTM transcript pairs (Table 3). The most significant *cis-*eQTM association showed cg16411857 (proximal to *NLRC5*) was significant with expression of *NLRC5, MT2A, NUP93*, and *CPNE2* (Table 2, lowest -log10pval, −35.5). *NLRC5* has been proposed for targeted therapy related to immune system regulation in central nervous disorders [56] and diabetic nephropathy [57]. Another novel gene identified in the current study, *CADM3* (cell adhesion molecule 3; cg09476997), beyond being associated with age (Table S8), has been associated with *MCP-1* (Monocyte chemoattractant protein 1) [58] which regulates monocyte and macrophage recruitment during an inflammation event [59]. In addition, *CADM3* is located on chr1:159,171,615–159,203,313, which is ~ 500kb from the *CRP* gene (chr1:159,712,289–159,714,589). Other novel CpG sites identified were previously associated with Crohn’s disease, inflammatory bowel disease, smoking, and age for *NALCN* (cg03185794) and *ZNF792* (cg24678320).

The results from this study show downstream implications of DNA methylation, including gene enrichment from inflammation pathways. Regulation of the NF-kB transcription factor is an established signalling pathways involved in the initiation and development of inflammatory disease [60], and was highlighted by the GO gene enrichment analyses for proximal genes and *cis-*eQTM CpG-gene transcript pairs (GO:0032088; Table 3; Table S6), and the KEGG pathway analysis (Fig. S1). Previously characterized inflammasome mediators, *AIM2* [60], *NLRC5* [54], and *NOD2* [61] were specifically enriched in this pathway. Positive regulation of innate immune response was indicated in the GO enrichment pathway using *cis-*eQTM transcript pairs (GO:0045089) (Table 3) and included *IFI16* and *IRF7* gene transcripts that were highly significant for the *cis-*eQTM analysis (Table 2). *IFI16* (significant with cg10636246, which is proximal to *AIM2*) activates inflammasome and type 1 interferon signalling pathways during the inflammatory process [62,63], and *IRF7* (significant with cg23570810, which is proximal to *IFITM1*) is an interferon that promotes inflammation and inflammatory disease [64]. Both *IFI16* and *IRF7* also contributed to the significant KEGG NOD-like receptor signalling pathway (Fig. S2b), and *IRF7* to the regulation of macrophage apoptotic process GO gene enrichment pathway (GO:20000109, FDR < 0.05; Table 3) and KEGG cytosolic DNA-sensing pathway (Fig. S2a). The CpG sites proximal to *IFI16* and *IRF7* were not significantly associated with CRP level (discovery meta-analysis p-values > 0.0003); however, cg20597486, for example, has been identified in rheumatoid arthritis associated differential methylation [65].

Our results show a causal association with the *PRKAR1B* gene for both the White and Black or African American MR analyses. Methylation of this CpG site has been previously associated with Crohn’s and inflammatory bowel disease, age, smoking, lipid levels, and BMI (Table S9) [66,67]. Another causal connection includes the *DCAF5* gene (cg06881965). DCAFs (DNA damage-binding protein Cul4-associated factors) are substrate-specific subunit proteins used by CRL4 ubiquitin E3 ligases to target specific proteins for degradation [68]. Recent findings indicate CRL4-DCAF5 targets methylated proteins for ubiquitin-dependent proteolysis [68] including SOX2 protein which is a master stem cell associated with many different types of cancers and has been proposed as an anticancer target [69,70]. Lastly, *IGSF6* is a novel member of the immunoglobulin superfamily with anti-tumour activity on macrophages and immune cell infiltration in cancer and inflammation tissue [71–73]. Genetic variation at distinct linkage disequilibrium groups within the *IGSF6* gene have been considered as a positional and functional candidate for inflammatory bowel disease, based on the proximity of this gene relative to the *NOD2* (also known as *CARD15*) gene which has reported a strong association with Crohn’s disease [74]. The causal association demonstrated here indicates a role of methylation at site cg02481950 in inflammation.

A major strength of this study is the racial and ethnic diversity, and the large sample size of well phenotyped participants. Comprehensive inclusion of covariates reduced the influence of confounding variables. These strengths allowed for power to detect CpG associations with inflammation biomarkers and to explore the generalizability of the findings across racially and ethnically diverse populations. Our study benefitted from available genetic and methylation data from WHI and ARIC participants. This allowed for the extraction of valid exposure instrument variables for the MR, calculated independently for each CpG site, adding precision in the instruments to evaluate causal direction. Past studies have had to rely on a single methylation score for all MR analyses which may overestimate the genetic effect sizes and/or mask effects at individual CpG sites [10,75], have been limited to the single source of mQTL data available in MR-Base (ARIES study, female only) [76], or reported low power due to limited availability of genetic data [9,77]. Lastly, the combination of epigenetics, eQTM analysis of CpG-transcript pairs, pathway enrichment analysis, and tests of direction of causality allowed for the exploration of functional properties of our findings.

In this study we assessed circulating inflammation biomarkers that are (generally) produced in tissues such as the liver and were used estimate the inflammation event in tissue of various organs. However, in the current study we could not estimate tissue-specific methylation changes due to availability of data. For CRP, this would underestimate the exposure from effects of the non-soluble form. The pleiotropic effects of CRP are a limitation for capturing the causal pathway associated with methylation, and interpretation of the results should take this into consideration [5]. Our cross-sectional design could not assess stability of findings over time; however our large sample size and replication data set support that DNA methylation profiles described in this paper are relevant to current levels of CRP. We also restricted analyses to loci present on the HM450 (Illumina) BeadChip so could not evaluate potentially important CpGs that are not measured on the array. White blood cells were used to measure epigenetic changes across all studies; we adjusted for white blood cell proportions. The cohorts used for this study represent a case-mix of participants. We adjusted or stratified for known case status (e.g., WHI substudy ba23 was stratified by case and control participants) but did obtain full information on chronic disease case mix, disease severity, and/or duration of disease from all participants. The adjustment for genomic inflation and test of heterogeneity following the meta-analysis would capture effects from unaccounted for disease, or heterogeneous effects from residual confounding from the white blood cell proportion estimates, on the overall association of differential methylation and CRP level. Alternatively, CRP associated sites may not be identified in the discovery stage due to high variance in effect estimates or would not replicate. CpG site replication may be further limited by sample size (i.e., statistical power) and non-paired samples [>1 year between methylation and CRP measurements for two studies (GENOA, *n* = 1202; AMISH, *n* = 288) (Table S1)]. The correlation of repeat CRP measurements over time has been shown to decrease for intervals >3 y [78,79]. Although the time interval in the GENOA and AMISH studies cannot be fully accounted for, a sensitivity analysis did show the retention of 107 of the 113 replicated CpG sites, including all five novel sites, when restricting the replication dataset to MESA and CHS with paired methylation and CRP samples (Table S4). Nonetheless, the number of replicated sites at a multiple testing corrected significance level may be restricted and CRP associated sites in the discovery dataset may be significantly associated with CRP, particularly those that have already been identified in prior papers (Table S4).

## Conclusions

CRP levels increase during infections and in chronic inflammatory disease linked to cardiovascular disease, cancer, and diabetes. The development of therapeutic strategies directed towards cellular processes associated with inflammation requires a deeper understanding of the molecular mechanisms. Evaluating the role of the epigenome in disease risk and along the causal pathway of these complex diseases could be valuable in understanding the specific cellular processes and pathogenic mechanisms, ultimately leading to more effective therapeutic targets across racially and ethnically diverse populations. This large-scale EWAS identified 113 CpG-CRP associations. Of the five novel sites identified in this study, *NLRC5* was previously identified as a potential treatment target for inflammation-related immune responses. In this study, we also provide insights on downstream pathways affected by DNA methylation, including the identification of the *cis*-eQTM CpG-gene transcript pairs *IFI16* and *IRF7* that were significantly enriched in both the innate immune response GO biological pathway and NOD-like receptor signalling KEGG pathway. Our MR analyses reported three causal CpG sites and showed *PRKAR1B* was causal when using instrumental variable from two race strata. In all, the inflammation-associated DNA methylation markers highlighted here could serve as indicators to measure low-grade inflammation. Forthcoming analyses will incorporate social determinant of health, sex/gender, lifestyle and behavioural factors, as well as genetic predictors such as a polygenic score that may further the understanding of the relationship of inflammation biomarkers and methylation (and genetic) profiles to improve detection, prevention, and treatment approaches for chronic illness.

## Supplementary Material

Supplemental Material

Supplemental Material

## Data Availability

Datasets used in this study are listed in Table 1. The data supporting the conclusions of this article are included within the article (and supplemental material). Data used for the analyses described in this manuscript are available on dbGaP under accession numbers phs000223 (ARIC), phs000220 (MEC), phs000974 (FHS), phs001416.v3.p1 (MESA), and phs000227 (WHI). Data for CHS can be requested at https://chs-nhlbi.org/node/6222. For studies not listed above, the data that support the findings of this study are available from the corresponding authors [L.R. (JHS), H.X. (AMISH), J.S. (GENOA)] upon reasonable request.
