# Supplementary material for: Methylation patterns associated with C-reactive protein in racially and ethnically diverse populations
Source: Epigenetics. 2024 Apr 3;19(1):2333668. doi: 10.1080/15592294.2024.2333668 (PMC10996836; doi:10.1080/15592294.2024.2333668)
Supplement: Supplemental Material [file KEPI_A_2333668_SM2936.docx]

**Supplemental Text**

**Methylation patterns associated with C-reactive protein in racially and ethnically diverse populations**

Lundin et al.

**Additional information on participating studies**

**WHI**

Women’s Health Initiative (WHI) data was comprised of four ancillary studies [EMPC (AS315), BA23, AS311, and Long Life Study (LLS)]. Epigenetic Mechanisms of PM-Mediated CVD Risk (WHI-EMPC) study assessed epigenetic mechanisms underlying associations between ambient particulate matter air pollution and cardiovascular disease within the WHI Clinical Trials (CT). From this population, DNA methylation was measured in 2200 randomly selected participants (Screening Visit,) (CT, *n* = 2200), and remeasured in 200 participants at a second visit (stage 2: Annual Visit (AV) 3, or AV6). Broad Agency Announcement 23 (WHI-BA23), also known as Integrative Genomics and Risk of CHD and Related Phenotypes in the Women's Health Initiative, was a case-control study assessing predictors of coronary heart disease (CHD) within the WHI CT (*n* = 1664) and observational study (OS, *n* = 442), where cases were identified using eight biomarkers of CHD. By design, WHI-BA23 oversampled African Americans and Hispanic or Latino/a Americans and required all participants to have undergone genome-wide genotyping and profiling of seven cardiovascular disease biomarkers. Ancillary Study 311 (AS311) was a matched case-control study of bladder cancer among women within the WHI CT (*n* = 405) and OS (*n* = 455) [1]. The LLS participants were CT and OS participants with follow-up visits ranging from 14 to 19 years after initial enrollment (hence the higher average age relative to the other studies); participants do not overlap with the those in the studies mentioned above. For all ancillary studies above, inflammation biomarkers were measured in whole blood samples collected within 1-year of the DNA methylation measurement.

**ARIC**

The Atherosclerosis Risk in Communities (ARIC) study included data from two ancillary studies of African Ancestry and European Ancestry [referred to in the main text as White]. ARIC is an ongoing prospective cohort study investigating the etiology of CHD in four US communities: Forsyth County, North Carolina; Jackson, Mississippi; Minneapolis, Minnesota; Washington County; Maryland. Participants were aged 45-64 and followed up in each community over 30 years with 7 study visits [2,3]. DNA methylation was measured in 2879 Black or African American and 1100 White participants from ARIC in visit 2 (1990–1992) or visit 3 (1993–1995). This study used the DNA methylation samples from visit 3 because of paired inflammation biomarker measurements at this exam. For the subjects that overlap between ARIC and JHS, all JHS participants were retained, after restrictions based on relatedness (second degree relatives defined as parent and child, or siblings as determined based on family IDs), and 435 from ARIC that overlapped with JHS were excluded.

**JHS**

The Jackson Heart Study (JHS) was initiated in 1998 as a longitudinal investigation of genetic and environmental risk factors associated with the disproportionate burden of cardiovascular disease in African Americans. This study is an extension of the ARIC study, with additional recruitment of Black or African American men and women, aged 35-84, in Jackson, Mississippi USA. JHS participants received three back-to-back clinical examinations (Exam 1, 2000-2004; Exam 2, 2005-2008; and Exam 3, 2009-2013) that have generated extensive longitudinal data on traditional and putative cardiovascular disease risk factors. DNA methylation and inflammation biomarkers data used for this study was all collected at baseline, and retained one individual per family group (comparing to parents and siblings). The JHS data for this study was obtained with a Data and Materials Distribution Agreement for JHS ASC# P1231.

**MEC**

The Multiethnic Cohort Study (MEC) is a prospective study of > 215,000 men and women of five racial and ethnic groups: Black or African American, Asian or Asian American (Japanese), Pacific Islander (Native Hawaiian), Hispanic or Latino/a, and White participants, recruited from the US state of Hawaii and from Southern California, primarily Los Angeles County, between 1993 and 1996 [4]. The MEC has a biorepository of blood specimens from approximately 70,000 participants, who provided samples around 10 years after cohort entry. For the present study, we focused our analysis on a subcohort of participants who had genomic ancestry, DNA methylation and CRP measured and were current smokers with no lung cancer history at the time of biospecimen collection [5]. For genomic ancestry and DNA methylation, individuals were selected as part of a subcohort of current smokers at time of blood draw who were genotyped using the Illumina Human1M-Duo BeadChip (1,199,187 single nucleotide polymorphisms [SNPs]) [6] and had DNA methylation measured by Illumina MethEPIC chip. High sensitivity CRP (hsCRP) was measured from fasting blood samples obtained between 1994 and 2016 [7].

**FHS**

The Framingham Heart Study (FHS) is a three-generation community-based cohort initiated in 1948 with recruitment of the original participants from Framingham, Massachusetts USA. The Framingham Offspring cohort was recruited in 1971, and consisted of 5124 participants who are the offspring and the spouses of offspring of the Original cohort [8]. Methylation and inflammation biomarker data was measured in peripheral blood sample collected from 2,846 offspring cohort participants who attended the eighth examination (2005–2008).

**MESA**

The Multi-Ethnic Study of Atherosclerosis (MESA) study is a longitudinal, population cohort study designed to examine risk factors for and the progression of CHD. Participants aged 45–84 years without clinically apparent CHD were recruited between July 2000 and August 2002 from six regions in the USA: Winston-Salem, North Carolina; Northern New York, New York; Baltimore, Maryland; St. Paul, Minnesota; Chicago, Illinois; and Los Angeles, California. DNA methylation was derived from peripheral blood mononuclear cell samples at Exam 1 or Exam 5 in a random sample of 1200 non-Hispanic White, Black or African American, Hispanic, and Chinese American participants [9]. This study used the DNA methylation samples from exam 1 because of paired inflammation biomarker measurements at this exam.

**AMISH**

The Old Order Amish (OOA) subjects included in this study were participants of several studies of cardiovascular health in relatively healthy volunteers from the OOA community of Lancaster County, Pennsylvania USA, and their family members. Participants aged 21-80 years at the time of recruitment. The subjects on whom the methylation chip was used were participants of the Heredity and Phenotype Interaction (HAPI) heart study [10,11], Amish Longevity Study [12], the Pharmacogenomics of Anti-Platelet Intervention (PAPI) study [13]. Inflammation biomarkers were measured in whole blood samples collected within 6-years of the DNA methylation measurement (range, 0-6.2 years; mean 2.5 years).

**GENOA**

The Genetic Epidemiology Network of Arteriopathy (GENOA) study is a community-based study of hypertensive sibships designed to investigate the genetics of hypertension and target organ damage in African Americans from Jackson, Mississippi and non-Hispanic whites from Rochester, Minnesota [14]. Initial recruitment (phase I: 1996-2001) included all members of sibships with ≥2 individuals with hypertension clinically diagnosed before age 60. DNA methylation was measured on peripheral blood leukocytes in Black or African American participants on either the HM450 (n=272) or EPIC chips (n=931). Inflammation biomarkers were measured on all participants at the second examination (phase II: 2001-2005), along with a subset of repeat sampling of DNA methylation (n=292). Only phase 1 methylation samples were used for this study. The time between DNA methylation and inflammation biomarker measurement was 1.8-7.5 years (mean. 5.2 years) for the EPIC chip data and 3.0-7.1 years (mean, 4.8 years) for the HM450 data.

**CHS**

The Cardiovascular Health Study (CHS) is a population-based cohort study of risk factors for coronary heart disease and stroke in adults ≥65 years conducted across four field centers: Forsyth County, North Carolina; Sacramento County, California; Washington County, Maryland; and Allegheny County, Pennsylvania [15]. The original predominantly White cohort of 5,201 persons was recruited in 1989-1990 from random samples of the Medicare eligibility lists; subsequently, an additional predominantly African American cohort of 687 persons were enrolled for a total sample of 5,888. DNA methylation and inflammation biomarkers were measured on a randomly selected subset of African American participants and White participants from study year 5 (1992-3). Although selection was determined for a case-control study on cardiovascular disease, only 12 of the 744 samples (<2%) used in this study were from CHD cases.

**References**

[1] WHI. Design of the Women’s Health Initiative clinical trial and observational study. Controlled Clinical Trials. 1998;19(1):61-109.

[2] ARIC. The Atherosclerosis risk in COMMUNIT (ARIC) study: design and objectives. American Journal of Epidemiology. 1989;129(4):687-702.

[3] Sharrett AR, Investigators A. The atherosclerosis risk in communities (ARIL) study introduction and objectives of the hemostasis component. Annals of Epidemiology. 1992;2(4):467-469.

[4] Kolonel LN, Henderson BE, Hankin JH, et al. A multiethnic cohort in Hawaii and Los Angeles: baseline characteristics. American Journal of Epidemiology. 2000;151(4):346-357.

[5] Murphy SE, Park S-SL, Thompson EF, et al. Nicotine N-glucuronidation relative to N-oxidation and C-oxidation and UGT2B10 genotype in five ethnic/racial groups. Carcinogenesis. 2014;35(11):2526-2533.

[6] Patel YM, Stram DO, Wilkens LR, et al. The Contribution of Common Genetic Variation to Nicotine and Cotinine Glucuronidation in Multiple Ethnic/Racial PopulationsGWAS of Nicotine and Cotinine Glucuronidation. Cancer Epidemiology, Biomarkers & Prevention. 2015;24(1):119-127.

[7] Morimoto Y, Conroy SM, Ollberding NJ, et al. Ethnic differences in serum adipokine and C-reactive protein levels: the multiethnic cohort. International Journal of Obesity. 2014;38(11):1416-1422.

[8] Kannel WB, Feinleib M, McNamara PM, et al. An investigation of coronary heart disease in families: the Framingham Offspring Study. American Journal of Epidemiology. 1979;110(3):281-290.

[9] Bild DE, Bluemke DA, Burke GL, et al. Multi-ethnic study of atherosclerosis: objectives and design. American Journal of Epidemiology. 2002;156(9):871-881.

[10] Mitchell BD, McArdle PF, Shen H, et al. The genetic response to short-term interventions affecting cardiovascular function: rationale and design of the Heredity and Phenotype Intervention (HAPI) Heart Study. American Heart Journal. 2008 May;155(5):823-8.

[11] Roy-Gagnon MH, Weir MR, Sorkin JD, et al. Genetic influences on blood pressure response to the cold pressor test: results from the Heredity and Phenotype Intervention Heart Study. Journal of Hypertension. 2008 Apr;26(4):729-36.

[12] Sorkin J, Post W, Pollin TI, et al. Exploring the genetics of longevity in the Old Order Amish. Mechanisms of Ageing and Development. 2005 2005/02/01/;126(2):347-350.

[13] Shuldiner AR, O'Connell JR, Bliden KP, et al. Association of cytochrome P450 2C19 genotype with the antiplatelet effect and clinical efficacy of clopidogrel therapy. JAMA. 2009 Aug 26;302(8):849-57.

[14] Daniels PR, Kardia SL, Hanis CL, et al. Familial aggregation of hypertension treatment and control in the Genetic Epidemiology Network of Arteriopathy (GENOA) study. The American Journal of Medicine. 2004;116(10):676-681.

[15] Fried LP, Borhani NO, Enright P, et al. The cardiovascular health study: design and rationale. Annals of Epidemiology. 1991;1(3):263-276.
